# The role of miRNA and lncRNA in gastric cancer

**DOI:** 10.18632/oncotarget.19197

**Published:** 2017-07-12

**Authors:** Ning-Bo Hao, Ya-Fei He, Xiao-Qin Li, Kai Wang, Rui-Ling Wang

**Affiliations:** ^1^ Department of Gastroenterology, General Hospital of the PLA Rocket Force, Beijing, China; ^2^ Intensive Medical Center, 302 Hospital of PLA, Beijing, China; ^3^ Department of Ophthalmology, General Hospital of the PLA Rocket Force, Beijing, China; ^4^ New Era Stoke Care and Research Institute, General Hospital of the PLA Rocket Force, Beijing, China

**Keywords:** gastric cancer, miRNA, lncRNA

## Abstract

Gastric cancer is one of the most common cancers and has the highest mortality rate worldwide. It is worthwhile to explore the mechanism of gastric cancer progression. An increasing number of studies have found that non-coding RNAs including miRNA and lncRNA play important roles in gastric cancer progression. This review summarized the role of ectopic miRNA in gastric cancer proliferation, growth, migration, invasion and apoptosis. Meantime, aberrantly expressed miRNA also received a great deal of attention as potential biomarker for gastric cancer diagnosis and therapy. Over the last decade, lncRNA was considered to regulate gastric cancer progression at the transcript and post-transcript level. At the transcript level, lncRNA induced gastric cancer progression by changing chromatin modification and mRNA stabilization to regulate mRNA and miRNA expression. Furthermore, lncRNA regulated gastric cancer progression by completely combining with miRNA to produce ceRNA or promote protein stabilization at the post-transcript level. Greater attention of miRNA and lncRNA in gastric cancer can provide new insight of mechanism of cancer development and may be acted as a new anticancer target.

## INTRODUCTION

Gastric cancer is the fourth most common cancer and the third leading cause of cancer mortality worldwide [1]. In China, the most recent statistics indicate that gastric cancer is second most common cancer in terms of incidence (679.1 per 100,000) and mortality (498.0 per 100,000) [2]. In U.S., it was estimated that 26,370 new cases of gastric cancer were diagnosed in 2016 with 10,370 estimated deaths [3]. In recent years, the 5-year mortality was significantly reduced for the early gastric cancer given the development of enterscopy and surgical technique. However, for advanced gastric cancer, the 5-year mortality remains 30% to 50% [4]. Advanced gastric cancer mortality is associated with peritoneal dissemination, hematogenous spread and lymph node metastasis. Thus, it was worthwhile to explore the mechanism of gastric cancer progression such as proliferation, growth, migration, invasion and apoptosis. Numerous studies have demonstrated that non-coding RNAs play important roles in gastric cancer progression [5–7]. In this study, we will review the role of non-coding RNA family in gastric cancer progression.

Non-coding RNA is an RNA molecule that cannot code protein. Based on the length, non-coding RNA are divided into two major classes. Large RNAs (greater than 50 nt) includes long non-coding RNA (lncRNA), small nucleolar RNA, circular RNA (ceRNA), tRNA and rRNA [8]. Small RNA (less than 50 nt) includes microRNA (miRNA), siRNA and piRNA [9]. Currently, numerous studies have found that miRNA and lncRNA play important roles in gastric cancer progression.

### miRNA involved in gastric cancer progression

miRNA is a highly conserved non-coding sequences that is generally 18 to 24 nucleotides in length [10–12]. First, miRNA is transcribed by RNA polymerase II and RNA polymerase III from introns, exons, intro-exon junctions or their own genes as pri-miRNA. After Drosha and DGCR8 processing in nucleus, pri-miRNA is transcribed as pre-miRNA and subsequently mature miRNA. Mature miRNA combines with other associated proteins to form the active RNA-induced silencing complex (RISC). RISC combines with the 5′UTR, ORF or 3′UTR of a target gene mRNA to suppress its translation or induce its degradation [13–16]. One miRNA can combined with several different mRNAs at several different points. A target gene mRNA can also combine with several miRNAs [12, 17].

### miRNAs in gastric cancer progression

To date, greater than 2500 miRNAs have been identified (miRbase database) and their ectopic expression is associated with tumor proliferation, invasion, metastasis, tumor growth and apoptosis. Both onco-miRNA and suppressing miRNAs play important roles in gastric cancer progression. Changes in miRNA expression and its role in gastric cancer are summarized in Table 1. For example, a previous study found that the onco-miRNA miR-130 was over-expressed in gastric cancer. The over-expressed miR-130 promoted gastric cancer proliferation and metastasis by combining with the 3′UTR of TGF-β and inhibiting its expression [18]. Our laboratory found that miR-1266, miR-1207-5p and miR-1182 exhibited relatively low expression in gastric cancer and promote cancer cell proliferation and metastasis. Further research found that miR-1266 and miR-1207-5p combine with the 3′UTR of hTERT to inhibit its expression and cancer cell proliferation and metastasis [19]. However, miR-1182 cannot combine with of the 3′UTR of hTERT. After a literature review and additional experiments, we found that miR-1182 inhibit hTERT expression by targeting its open reading frame (ORF) regions [20]. This result indicates that miRNA always combine with its target gene 3′UTR. However, the miRNA may occasionally combine with the ORF region. For tumor growth, a previous study found that over-expressed miR-24 can promote gastric cancer growth and suppress apoptosis by inhibiting BCL2L11 expression [21]. Zhang et al. found that Sirt7 can significantly inhibit miR-34a expression in gastric cancer and ultimately promote tumor growth and inhibit apoptosis [22]. Previous studies found that miRNA clusters (miR-15b, miR-16, miR-34, miR-181b, miR-181c, and miR-497) exhibit relatively low expression in gastric cancer and promote the expression BCL-2 which inhibits apoptosis [23]. A cluster of onco-miRNAs (miR-25, miR-93, miR-106b, and miR-130) is overexpressed in gastric cancer and inhibits gastric cancer cell apoptosis by suppress Bim expression. Since Bim is considered as a pro-apoptosis protein [24].

**Table 1:** The ectopic expression of miRNA in gastric cancer

miRNA	Relative expression	Target mRNA	Cell biology	Clinicopathologic feature	Reference
miR-17-5p/20a	Up	TP53INP1	Cell proliferation and apoptosis	Tumor size	Wang et al. [25]
miR-100	Down	ZBTB7A	tumor growth, invasion and metastasis	lymphatic metastasis	Shi et al. [26]
miR-125b	Up	PPP1CA	cellular proliferation, migration, and invasion	tumor size and depth of invasion, lymph nodes, distant metastasis, TNM stage and poor prognosis.	Wu et al. [27]
miR-133b	Down	PTBP1	Tumor growth	No association with the clinicopathological future	Sugiyama et al. [28]
miR-145	down	Ets1	migration, invasion, and angiogenesis	tumor invasion and metastasis	Zheng et al. [29]
miR-148a	Down	ROCK1	Migration and invasion	TNM stage and lymph node-metastasis	Zheng et al. [30]
miR-196a/-196b	Up	radixin	cell migration and invasion	TNM stage, lymph node metastasis, poor prognosis	Tsai et al. [31]
miR-199a-5p	Up	klotho	cell migration and invasion	Tumor size, TNM stage and lymph node metastasis	He et al. [32]
miR-302	Down	IL-8	Migration and invasion	metastasis and prognosis	Chen et al. [33]
miR-506	Down	ETS1	Angiogenesis and EMT	Poor prognosis	Li et al. [34]
miR-940	Up	ZNF24	Migration and invasion	poor prognosis	Liu et al. [35]
miR-1182	Down	hTERT	cell proliferation, migration and invasion	Tumor size, differentiation, metastasis, TNM Stage and poor prognosis	Zhang et al. [20]
miR-1207-5p/miR-1266	Down	hTERT	cell proliferation, migration and invasion	Tumor size, differentiation, metastasis, TNM Stage and poor prognosis	Chen et al. [19]
miR-29a/c	Down	VEGF	vascular cell growth, metastasis, and tube formation	-	Zhang et al. [36]
miR-29b/c	Down	DNMT3A	Migration and invasion	Invasion	Cui et al. [37]

### miRNA in gastric cancer diagnosis

Tumor diagnosis is an important process that may influence the risk of progression, recurrence and death. However, early detection methods or screening methods must be explored for various types of cancers. Thus, multiple studies are focused on exploring the biomarkers for cancer detection and progression. Numerous miRNAs are aberrantly expressed in the plasma and serum of gastric cancer patients [6, 38–41]. For example, miR-223, miR-233, miR-378, miR-421, miR-451, miR-486-5p and miR-199-3p are over-expressed in sera of gastric cancer patient [42–45]. Wang and colleagues found that miR-233 was over-expressed in gastric cancer patient serum at a normalized cutoff of 0.21. miR-233 yields a receiver operating characteristic (ROC) area under the curve (AUC) of 0.85, a sensitivity of 81% and specificity of 78% [42]. The level of miR-233 expression in patient serum was positively associated with tumor differentiation grade, TNM stage, tumor size and metastasis status [42]. Wu and colleagues found that miR-421 was overexpressed in 90 cases of gastric cancer patient sera compared with 90 controls with an AUC, sensitivity and specificity of 0.821, 95.5% and 89.1%, respectively. These values were increased compared with CA125 and CEA for gastric cancer detection. The high expression of miR-421 in mononuclear cells acts as a biomarker for gastric cancer circulating tumor cells, which may be used for early diagnosis for gastric metastasis [43]. Furthermore, *in vivo* and *in vitro* experiments demonstrated that the onco-miR-421 promotes tumor proliferation, invasion and metastasis [46]. Jiang et al. also found that miR-421 was overexpressed in gastric cancer tissues but had no significant association with the clinic-pathological feature. [47].

In contrast, let-7a, miR-375, miR-20a-5p and miR-320 expression was relatively reduced in gastric cancer patient serum [48, 49]. A previous study demonstrated that let-7a exhibited relatively low expression in plasma of gastric cancer patient compared with healthy controls, whereas the expression of miR-17-5p, miR-106a, miR-106b and miR-21 was significantly elevated in gastric cancer plasma [50]. Further analysis found that the miR-106a/let-7a ratio in 69 gastric cancer patients and 30 controls revealed a maximum AUC of 0.879, with a sensitivity of 85.5 and specificity of 80.0% [51]. Tang and colleagues also found that let-7a was suppressed in gastric cancer tissue. *In vivo* and *in vitro* experiments showed that let-7a overexpression significantly suppressed PMK2 expression, which inhibited tumor proliferation, migration and invasion [52]. Previous studies demonstrated that miR-375 was suppressed in gastric cancer. Overexpression of miR-375 suppresses gastric cancer progression by targeting p53, JAK2, ERBB2 and STAT3 [53, 54]. Zhang and colleagues found that miR-375 expression was significantly suppressed in both distal gastric adenocarcinoma tissues and patient serum compared with healthy controls. At a normalized cutoff of 0.218, the AUC of serum miR-375 was 0.835 with a sensitivity of 85% and specificity of 80% [48]. These studies indicate miRNA severs as an interesting diagnostic biomarker. However, large-scale clinical research is needed to demonstrate that miRNA can serve as a diagnostic biomarker for gastric cancer.

### miRNA in gastric cancer therapy

Recent studies have found that miRNA-based cancer therapy may be a promising and challenging path. On one hand, miRNA-based drugs that overexpress the suppressed-miRNA or inhibit the onco-miRNA can inhibited tumor progression by suppressing relative signal pathway [40, 55]. For example, miR-34 suppresses miRNA in a few tumors including breast cancer, liver cancer, lung cancer and gastric cancer [56–58]. Recently, a clinical trial of MRX34 (Mirna Therapeutics, TX, USA) constructed a miR-34 mimetics to restore miR-34 expression in cancer cells. The agent was used to treat liver cancer and liver metastasis of other cancers in phase I clinical trial protocol [40, 59]. Meantime, in a pre-clinical study of non small cell lung cancer, MRX34 treatment significantly reduced the expression of the checkpoint signal PD-L1 and increase the infiltrating CD8+ cells in tumor tissues [60]. However, the MRX34 clinical trial was stopped in 2016 since multiple serious immune-related side effects were observed in patients. In detail, one patient receiving MRX34 experienced a prolonged partial response and lasted 48 weeks. Meantime, six others presented with stable disease of the total 47 patients. So the safety of MRX34 still needs for further research [61]. For gastric cancer, Ji and colleagues found that restoration of miR-34 significantly inhibits gastric cancer tumorspheres by promoting expression of tumor suppressing mutant p53 [62]. The miR-34 family targeted the 3′UTR of Yin Yang1 to inhibit its expression, reduced tumor growth and metastasis [63].

On the other hand, miRNA also played critical roles in drug resistance. An increasing number of studies have demonstrated that miRNA can significantly influence drug transporters, drug-metabolizing enzymes, transcription factor and nuclear receptors. Yang and colleagues found that miR-21 was over expressed in the cisplatin resistant cell line SGC7901. Knock-down of miR-21 expression can significantly increase the anti-proliferative effects and apoptosis induced by cisplatin [64]. Xia et al. found that miR-15/miR-16 family was significantly decreased in multidrug resistance (MDR) of gastric cancer cells. Over expression of miR-15 dramatically reduces the expression of BCL2, which reverses the MDR in gastric cancer cells by modulating cell apoptosis [65]. However, some problems should be considered, as one miRNA can target multiple genes and signaling, the off-target effect is unexpected. Thus based on miRNA therapy system should be explored with better specificity [36].

In total, we discussed the role of miRNA in gastric cancer progression. Both onco-miRNA and suppressing miRNA play critical roles in gastric cancer proliferation, growth, metastasis and apoptosis. The overexpression of miR-421 in the serum of gastric cancer patients may act as an early diagnostic marker. Meantime, overexpression of miR-34 significantly inhibited cancer progression in a few cancers and miR-34 may be used for cancer therapy in the future.

### The role of lncRNA in gastric cancer

In contrast with miRNA, lncRNA is an RNA transcript longer than 200 nt with no capacity for coding protein. Based on their location and orientation, lncRNAs are classified as intronic lncRNAs, intergenic lncRNAs, pseudogenes, sense or antisense transcripts, and retrotransposons [66, 67]. Functionally, lncRNAs regulate gene expression at any level, including chromatin modification, transcription, and post-transcriptional processing. A large number of studies have found that lncRNAs played important roles in numerous diseases and disease processing, including cardiovascular and cancer progression [68–71]. Recently, studies have found that the ectopic lncRNA expression, including that of H19, TUSC7, MEG3 and MALAT1 significantly regulate gastric cancer cell proliferation, cell cycle, apoptosis, invasion, migration, metastasis, and tumorigenicity [7, 51, 72–74]. In this section, we will summarize the roles of lncRNA in gastric cancer progression.

### lncRNA regulates gene expression at the transcriptional level

**Figure 1:**
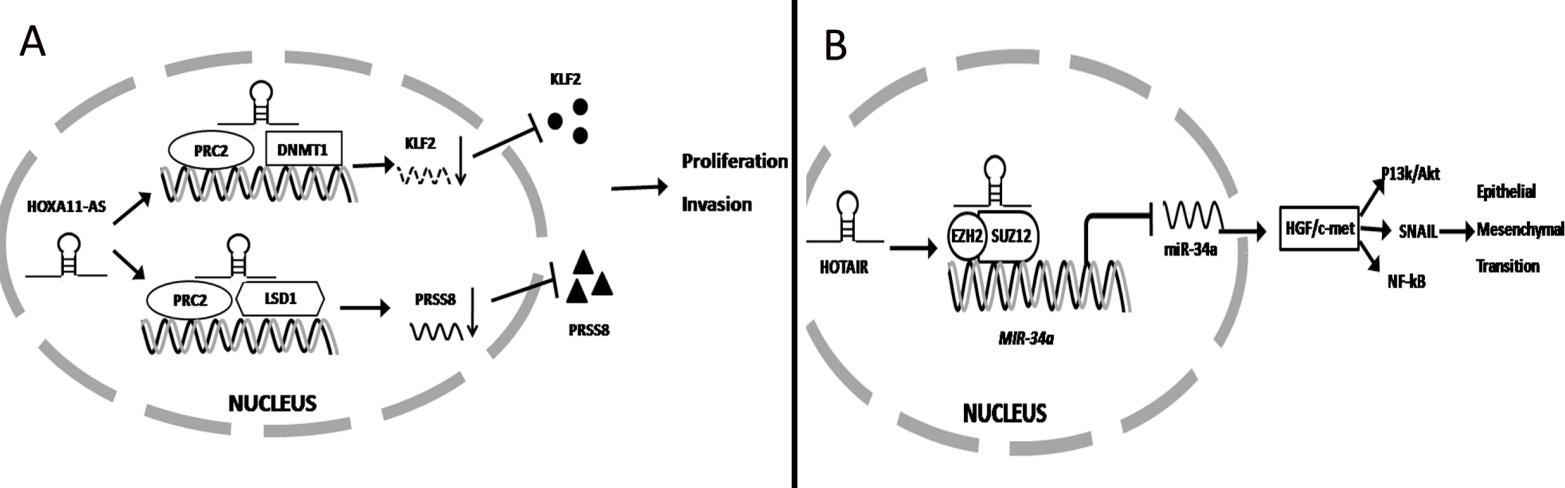
lncRNA regulates gene expression at the transcriptional level (**A**) HOXA11-AS binds to several RNA binding proteins (PRC2, LSD1 and DNMT1) and function as a scaffold to inhibit PRSS8 and KLF2 at the transcriptional level to promote gastric cancer proliferation and invasion. (**B**) HOTAIR binding to PRC2 significantly inhibited miR-34a expression at the transcript level ultimately activating HGF/c-met signaling to promote EMT.

Previous studies have demonstrated that 38% of lncRNA cooperate with at least one of multiple histone-modifying complexes, inducing DNA methylation and chromatin modification that ultimately leads to silence the expression of target mRNA [75, 76]. For example, Sun and colleagues discovered that only lncRNA HOXA11-AS was specifically over-expressed in gastric cancer but not in other cancers using the sequencing data from the TCGA project and microarray profile from GEO. High expression of lncRNA HOXA11-AS in gastric cancer patients was associated with poor prognosis. Knockdown of HOXA11-AS expression significantly reduces gastric cancer cell proliferation and promotes apoptosis. FISH and subcellular fractionation experiments demonstrated that HOXA11-AS was more prevalent in the nucleus. Another study found that HOXA11-AS acts as a scaffold to reduce KLF2 and PRSS8 expression at the transcriptional level, given that it can directly bind to the RNA binding proteins PRC2, LSD1 and DNMT1. Finally lncRNA-mediated assembly of PRC2 and LSD1 coordinates targeting of PRC2 and LSD1 for coupled H3K27 methylation and H3K4 demethylation [77]. (Figure 1A) In contrast, Xu et al. found that low expression lncRNA FENDRR in gastric cancer patients was associated with poor survival. Over-expression of FENDRR can significantly reduce gastric cancer metastasis [78]. FENDRR combines with PRC2 to form FENDRR/PRC complex, which inhibits MMP2/9 expression [78]. Sun et al. also found that lncRNA GClnc1 was significantly over-expressed in gastric cancer. Mechanistically, GClnc1 binds to WDR5 and KAT2A histone acetyltransferase, acts as a modular scaffold of WDR5 and KAT2A complexes, coordinates their localization and consequently alters gastric cancer proliferation, migration and invasion [79].

Recently, it was found that lncRNA epigenetically silences miRNA expression at the transcript level to promote gastric cancer progression. Previous studies have found that over-expression of lncRNA HOTAIR contributed to gastric cancer development and predicted a poor prognosis. Liu and colleagues further demonstrated that HOTAIR combined with EZH2 and SUZ12 to form a complex that directly binds to the miR-34a promoter to silence its expression via H3K27me3 modification. Finally, low expression of miR-34a significantly promotes HGF/c-met activation, which induces SNAIL, PI3K/Akt and NF-kB signaling to promote cancer development [80]. (Figure 1B) Zhang et al. reported that over-expression of lncRNA ANRIL was significantly associated with gastric cancer progression and can serve as an independent predictor for patient survival. Mechanistically, E2F1 directly binds to the ANRIL promoter to induce its expression. Then high expression of ANRIL combined with PRC2 significantly silences miR-99a/miR-449a expression at the transcriptional level, which ultimately increase mTOR, CDK6, and E2F1 expression. miR-99a/miR-449a binds to the 3′UTR of mTOR, CDK6, and E2F1 to inhibit their expression. In total, high expression of E2F1 induced ANRIL expression. Then the ANRIL/PRC2 complex inhibited miR-99a/miR-449a expression and promoted mTOR, CDK6 and E2F1 expression. Conversely, E2F1 promoted ANRIL expression which formed a positive feedback loop to promote gastric cancer cell proliferation [81].

lncRNAs also regulate target gene expression by directly interacting with its mRNA. For example, Xu et al. found that high expression of nuclear factor SP1 which is significantly expressed in gastric cancer, binds to the promoter of lncRNA TINCR to promote its expression. Silencing the expression of TINCR significantly reduces cancer cell proliferation, tumorigenicity and apoptosis. Mechanistic analyses indicated that TINCR was mostly present in the cytoplasm. RNA IP and pull-down assay showed that TINCR combined with STAU1 acts as a staufen-mediated mRNA decay (SMD) factor. Furthermore RNA immunoprecipitation (RIP) and RNA pull down assay showed that TINCR/STAU1 complexes directly interact with KLF2 mRNA, decrease KLF2 mRNA stability, and inhibit its protein expression. Subsequently, reduced KLF2 expression decreases CDKN2B/P15 and CDKN1A/P21 transcripts which finally promote cancer cell proliferation, migration, invasion and tumorigenicity [82]. In contrast, Yang and colleagues found that lncRNA GHET1 was significantly over-expressed in gastric cancer patients compared with healthy controls. Over-expression of GHET1 in MKN45 and AGS of gastric cancer cells significantly promotes cell proliferation *in vitro*. *In vivo* experiments showed that over-expression of GHET1 in MKN45 cells significantly promotes growth of xenograft tumors in nude mice. Mechanistically, RIP and RNA pull-down assay first showed that GHET1 can directly combine with insulin growth factor2 binding protein 1 (IGF2BP1). RIP assay and qRT-PCR results showed that IGF2BP1 combined with the mRNA of c-myc. Together, these results showed that lncRNA GHET1/IGF2BP1 complex combines with c-myc mRNA to increase its stability and promote protein expression. Finally, high expression of c-myc promoted gastric cancer cell proliferation [72].

### lncRNA regulates gene expression at the post-transcriptional level

**Figure 2:**
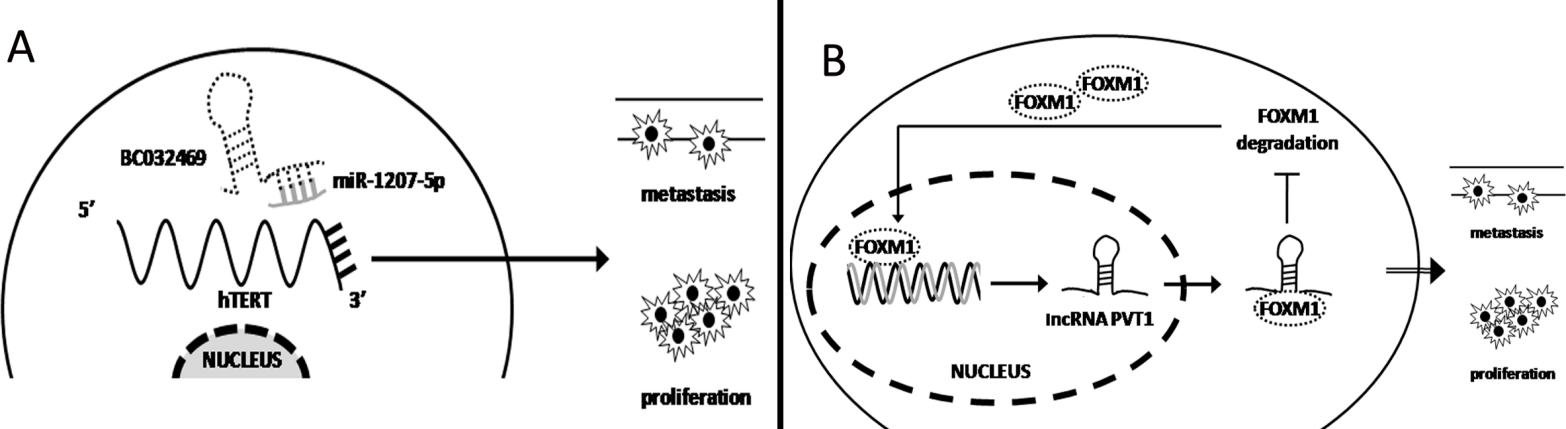
lncRNA regulates gene expression at the transcriptional level (**A**) lncRNA BC032469 functions as a ceRNA to sponge for miR-1207-5p, promoting hTERT expression to induce gastric cancer cell proliferation and metastasis. (**B**) FOXM1 induced lncRNA PVT1 expression at the transcriptional level. Then PVT1 binds to FOXM1 protein to increase its stabilization, forming a positive feedback loop and promoting gastric cancer cell proliferation and metastasis.

As previously described, miRNA regulates target gene expression at the post-transcriptional level. Thus, high expression of lncRNA can competitively combine with miRNA, acting as a sponge to induce miRNA disability and promote cancer progression. Our laboratory previously demonstrated low miR-1207-5p expression in gastric cancer. Over-expression of miR-1207-5p significantly inhibits cancer cell proliferation and metastasis by combining with the 3′UTR of hTERT to inhibit its expression. Recently, the further research found that lncRNA BC032469 expression was positively associated with hTERT expression and significantly promotes gastric cancer cell proliferation and metastasis. Mechanistically, RIP and Northern blot demonstrated that BC032469 directly binds to miR-1207-5p. Thus, BC032469 functions as a ceRNA to impair miR-1207-5p-dependent hTERT down-regulation, suggesting that it may a poor prognostic marker for gastric cancer [83]. (Figure 2A) Furthermore, Liu et al. found that over-expression of lncRNA HOTAIR in gastric cancer competes with miR-331-3p expression, which functions as a ceRNA to promote HER expression and promote gastric cancer progression [84].

lncRNA also regulate protein stabilization at the post-transcriptional level to promote gastric cancer progression. Xu and colleagues demonstrated that the promoter of lncRNA PVT1 contains binding sites for the transcriptional factor FOXM1. High FOXM1 expression in gastric cancer significantly induces PVT1 expression. Furthermore, high expression of PVT1 did not influence FOXM1 mRNA expression but can reversely bind to FOXM1 protein to increase its stabilization and inhibit 26s proteasome-mediated degradation. Thus, high expression of FOXM1 and PVT1 form a positive feedback loop that promotes gastric cancer proliferation and metastasis [85]. (Figure 2B).

In summary, lncRNA significantly regulates gene expression both in the nucleus and cytoplasm. In the nucleus, lncRNAs bind the polycomb group protein (PcG) complex, induce histone trimethylation and regulate relative gene mRNA expression at the transcriptional level. Meantime, lncRNAs directly bind with the promoter to regulate gene expression. In the cytoplasm, lncRNA also directly bind to mRNA to influence its stability and expression at the transcriptional level. Meantime, cytoplasmic lncRNA can regulate gene expression at the post-transcriptional level. As previously described, lncRNAs act as a sponge with miRNA and induce a ‘ceRNA’ to regulate gene expression. On the othe hand, lncRNA also influence protein stability and inhibit expression at the post-transcriptional level. Finally, we summarized the ectopic expression of lncRNA and its target gene and role in gastric cancer progression in Table 2.

**Table 2:** The ectopic expression of lncRNA in gastric cancer

lncRNA	Relative Expression	Molecular Mechanism	Cell biology	Clinicopathologic feature	Reference
LINC00673	up	functioning as a scaffold for LSD1 and EZH2 and repressing KLF2 and LATS2 expression	Proliferation, tumor growth, invasion, metastasis and apoptosis	Tumor size, TNM Stage, lymphatic metastasis and poor prognosis	Huang et al. [86]
SNHG5	down	Sponge with miR-32 to regulate KLF4 expression	Proliferation and migration	negatively associated with miR-32	Zhao et al. [87]
LincRNAFEZF1-AS1	up	functioning as a scaffold for LSD1 and repressing p21 by inducing H3K4me2 demethylation	Proliferation, tumor growth and apoptosis	Tumor size, TNM Stage and poor prognosis	Liu et al. [88]
PVT1	up	PVT1 directly bound with FOXM1 protein to increase its stability, FOXM1 reversely bound to PVT1 promoter to promote its expression	Proliferation and metastasis	Tumor size, distant metastasis and poor prognosis	Xu et al. [89]
lncRNA-GHET1	up	GHET1 combined with IGF2BP1 protien up-regulates c-Myc by increasing c-Myc mRNA stability	proliferation	Tumor size, invasion and poor prognosis	Yang et al. [72]
TINCR	up	TINCR binds to STAU1 down-regulate KLF2 expression by influence its mRNA stability	Proliferation and apoptosis	TNM stage and lymphatic metastasis	Xu et al. [82]
ANRIL	up	ANRIL binds with PRC2 decreased miR-99a/miR-449a expression by interact with its promoter	proliferation	Tumor size, TNM stage and poor prognosis	Zhang et al. [81]
LincHOTAIR	up	HOTAIR silenced miR34a expression by recruiting PRC2 and finally activated HGF/c-Met /Snail pathway to promote gastric cancer EMT	Invasion and metastasis	Invasion depth, lymphatic metastasis and poor prognosis	Liu et al. [80]
GAPLINC	up	GAPLINC sponge with miR211-3p to regulate CD44 associated downstream signal	Proliferation and Invasion	Tumor size, lymphatic metastasis, TNM stage and poor prognosis	Hu et al. [90]
FENDRR	down	decreased FENDRR expression induces FN1 expression and activates MMPs family	Migration and Invasion	Invasion depth, TNM Stage, lymphatic metastasis and poor prognosis	Xu et al. [78]
H19	up	H19 directly binds with ISM1 to regulate its expression	proliferation, migration, invasion and metastasis	TNM Stage, lymphatic metastasis and poor prognosis	Li et al. [91]
FER1L4	down	*FER1L4* liberated miR-106a-5p, downregulated *PTEN* expression	Proliferation and cell cycle	Tumor size, lymphatic metastasis, TNM stage and invasion depth	Xia et al. [92] Liu et al. [93]
GAS5	down	lncRNA GAS5 reduces the YBX1 protein and subsequently decreases YBX1-transactivated p21 expression	Cell cycle	-	Liu et al. [94]
nc886	down	nc886 knockdown activation of oncogenic FOS, NF-κB, and MYC as well as other pathways	proliferation	Poor prognosis	Lee et al. [95]
HOXA11-AS	up	HOXA11-AS functions as a scaffold for EZH2 and LSD1, HOXA11-AS acts as a ceRNA for miR-1297	Proliferation, cell growth, migration, invasion, and apoptosis	Tumor size, lymphatic metastasis, TNM stage and poor prognosis	Sun et al. [77]
GClnc1	up	GClnc1 bound WDR5 and KAT2A histone acetyltransferase, acted as a modular scaffold of WDR5 and KAT2A complexes, specified the histone modification pattern	proliferation, migration and invasion	Tumor size, Vascular metastasis, TNM stage and poor prognosis	Sun et al. [79]
BC032469	up	BC032469 bind to miR-1207-5p and effectively functioned as a sponge to modulate the derepression of hTERT	proliferation	Tumor size, poor proliferation and poor prognosis	Lu et al. [83]

## CONCLUSIONS

In this study, we discussed the role of miRNA and lncRNA in gastric cancer progression. Most miRNA bind to the 3′UTR of the target gene to regulate its expression and new research found that miRNAs also bind to the ORF region of target genes to influence expression. This information may broaden our understanding of whether miRNA can directly bind to the 5′UTR of target gene. Most studies have focused on the role of miRNA in cancer progression and the target gene of miRNA in the downstream. But few studies reported that which gene can induced the miRNA ectopic expression, so this part need to further research.

We summarized that lncRNA significantly binds to miRNA, mRNAs and proteins in gastric cancer, regulating gene expression at both the transcriptional and post-transcriptional levels. However, research on lncRNA in normal gastric cells is relatively limited. In the future, more research will focus on the role of lncRNAs in gastritis, atypical hyperplasia and early carcinoma. We hope that diagnosis and treatment based on lncRNAs will be realized in the future.
